# Calculated initial parenteral treatment of bacterial infections: Bacterial gastrointestinal infections

**DOI:** 10.3205/id000050

**Published:** 2020-03-26

**Authors:** Reinier Mutters, Peter Walger, Christoph Lübbert

**Affiliations:** 1 Institut für Medizinische Mikrobiologie und Krankenhaushygiene, Philipps-Universität Marburg, Germany; 2 Verbund Katholischer Kliniken Düsseldorf, Zentralbereich Hygiene, Infektionsmanagement und ABS, Düsseldorf, Germany; 3 Klinik und Poliklinik für Gastroenterologie und Rheumatologie, Universitätsklinikum Leipzig, Germany

## Abstract

This is the fourteenth chapter of the guideline “Calculated initial parenteral treatment of bacterial infections in adults – update 2018” in the 2^nd^ updated version. The German guideline by the Paul-Ehrlich-Gesellschaft für Chemotherapie e.V. (PEG) has been translated to address an international audience.

Bacterial gastrointestinal infections are still the leading cause of death worldwide. The chapter describes the etiology of bacterial gastrointestinal infections in Germany and their frequency. Recommendations are given for the calculated therapy of these infections and for targeted antibiotic therapy for known pathogens. Particular attention is paid to *Clostridium difficile*. The diagnostic and therapeutic options of antibiotic therapy of the various infection patterns in this pathogen are discussed.

## Zusammenfassung

Dies ist das vierzehnte Kapitel der von der Paul-Ehrlich-Gesellschaft für Chemotherapie e.V. (PEG) herausgegebenen S2k Leitlinie „Kalkulierte parenterale Initialtherapie bakterieller Erkrankungen bei Erwachsenen – Update 2018“ in der 2. aktualisierten Fassung.

Bakterielle gastrointestinale Infektionen gehören weltweit immer noch zu den häufigsten Todesursachen. Im Kapitel werden die Ätiologie bakterieller gastrointestinaler Infektionen in Deutschland und ihre Häufigkeiten beschrieben. Es werden Empfehlungen zur kalkulierten Therapie dieser Infektionen und zur gezielten Antibiotika-Therapie bei bekanntem Erreger gegeben. Besonderes Augenmerk wird auf *Clostridium difficile* gerichtet. Die diagnostischen und therapeutischen Optionen der antibiotischen Therapie der verschiedenen Infektionsbilder bei diesem Erreger werden diskutiert.

## Introduction

Bacterial gastrointestinal infections are still among the leading causes of deaths worldwide. According to WHO estimates, each year 1.7 billion people develop diarrhea and 760,000 children die from the effects of diarrhea [1]. In the vast majority of diarrheal diseases, an infection is the cause. Despite high hygienic standards and available treatments, infectious diarrheal diseases are among the most common infections even in Germany. However, they are usually easily managed. An epidemiological study found approximately 65 million cases of gastrointestinal illnesses per year in adults in Germany, corresponding to an incidence of 0.95 episodes per person/year. Illness duration averages 3.7 days, with 78% of the illnesses being associated with diarrhea, 12% with vomiting and 10% with both symptoms [2]. Other symptoms include nausea, stomach aches and, in some cases, fever. Blood and mucus in the feces and increased pain and fever are indications of an invasive pathogen, with the clinical transitions to enterocolitis or colitis being fluid. The pathogens are usually viruses and bacteria, in Germany parasites (in particular protozoa) are also less likely. 

Less than 40% of those affected receive medical treatment on an out-patient basis, less than 3% are hospitalized [3]. WHO distinguishes three clinical entities: watery (secretory) diarrhea, including cholera; bloody diarrhea; and persistent diarrhea lasting more than 14 days.

Bacterial gastrointestinal infections are usually caused by enterobacteria and other Gram-negative pathogens, by toxin-producing Gram-positive or Gram-negative pathogens or by toxin-producing anaerobes. Infections with enterotoxin-producing *Staphylococcus* *aureus* strains (classic clinical picture of “food poisoning” with immediate onset of symptoms (<4–6 hours) of symptoms after consumption) are most prevalent, as are *Campylobacter*, Salmonella and respiratory *Escherichia* *coli* infections [4]. Non-bacterial causes include mainly noro- and rotaviruses. Illnesses are significantly less often caused by *Giardia* *lamblia*, *Cryptosporidium* *parvum* or for example *Entamoeba* *histolytica*. Vibrios of the species *parahaemolyticus* and *cholerae* are rare (see Table 1 (Tab. 1)).

In recent years, the number of hospital admissions of acute infectious diarrheal illnesses per year has more than doubled in the group of over-65-year-olds. This is mainly due to the increase in colitis caused by toxin-producing strains of *Clostridium* *difficile*, with death rates having increased 10-fold [2].

Infections with enteropathogenic pathogens show different progressions. The majority are secretory diarrheas without signs of systemic inflammation (cholera-like). Invasive infections with blood and mucus in the feces as well as fever (dysentery) and, depending on the pathogen, various extra-intestinal manifestations or bacteriemic seeding can lead to severe, life-threatening progressions with hospitalization and fatal outcomes. The cause is either the production of toxins or intestinal invasion or adhesion of the pathogens. The infection route is usually consumption of plant- or animal-based food contaminated with bacteria but also of unclean drinking water. Direct contact with infected animals plays a rather minor epidemiological role. Nosocomial transmission can lead to outbreaks in hospitals and other health care facilities. The selection pressure of antibiotics is one of the major causes of the spread of Clostridium difficile infections. 

According to the Infection Protection Act (IfSG), there is a requirement to report infections with certain pathogens (see Table 2 (Tab. 2)). According to §6 (1) IfSG, even suspected cases and actual cases of microbial food poisoning or acute infectious gastroenteritis must be reported where this affects patients working in the food sector (§42 Sect.1) and cases of two or more similar illnesses where a single epidemiological cause is suspected (outbreak). There is a special reporting obligation for severe cases of Clostridium difficile if one of the following four clinical criteria for a severe progression is met:

1. Admission for treatment of community-acquired *Clostridium* *difficile* infection in a medical facility.
2. Transfer to an intensive care unit for treatment of CDI or its complications.
3. Surgery (colectomy) due to megacolon, perforation or refractory colitis.
4. Death <30 days after diagnosis and CDI as a cause or illness contributing to the death and/or detection of *Clostridium* *difficile* PCR ribotype 027.

In Austria, according to the Law on Epidemics, since 2010 CDIs with severe progression must be reported. Severe progression here constitutes the need for intensive care treatment, surgical intervention for a CDI or lethal progression of a CDI. 

## Diagnostics

Correct acquisition of a stool sample is a prerequisite for meaningful microbiological diagnosis. For the microbiological lab, patient history and clinical data (for example, diarrhea, possibly with blood/mucus, persistent diarrhea, stay abroad, antibiotic treatment) are important for the targeted isolation of enteritis/colitis pathogens. In general, the result of a single negative stool sample does not fully exclude relevant pathogens. The sample material in a quantity of approx. 2–3 spoonfuls of stool (if possible from different places in the feces) should reach the lab in a timely manner, in order to avoid overgrowth by the accompanying flora. Also, some pathogens die quickly and toxins are deactivated (for example *Shigella*, *Campylobacter*, *Clostridium* difficile toxin). In cases of suspected *Clostridium* *difficile/Clostridium* *perfringens* infection, examining multiple stool samples increases the sensitivity of toxin detection. Detection of bacteria is usually carried out via culture, in suspected cases of Salmonella by means of additional blood culture and toxin detection (as in the case of illnesses caused by *Staphylococcus* *aureus)* from the suspected food or, as in Shigella (Shiga toxins I and II), from bacterial cultures via PCR and immunoassays (EIA).

Diagnosis in case of suspected infection with *Clostridium* *difficile* (CDI), which currently can no longer be regarded as being acquired nosocomially alone, is usually done via direct detection of toxins A and/or B. The quick test for the pathogen’s GDH (glutamate dehydrogenase), which is also available commercially in combination with the toxin test, allows the detection of *Clostridium* *difficile* regardless of its toxigenicity and increases the reliability of rapid detection as a combination test. For typification of isolates, elaborate bacteriological anaerobic culture with subsequent ribotyping of the isolate in special labs is still necessary [5].

## Treatment

Fluid and electrolyte replacement is the basic treatment for gastrointestinal infections. There are three severity levels of dehydration: early dehydration with no other symptoms or findings; moderate dehydration with clinical signs of diminished skin turgor or elasticity, thirst and delirium and severe dehydration with shock, clouding or loss of consciousness and acute renal failure. Acute substitution of fluid loss can be achieved by oral rehydration solutions according to WHO recommendations (formula: 13.5 g of glucose, 2.9 g of sodium citrate, 2.6 g of sodium chloride, 1.5 g of potassium chloride per liter of water) or under in-patient treatment through parenteral administration of fluids and electrolytes. The target of rehydration is to avert exsiccosis, hypovolemic hypotension (volume deficiency shock), pre-renal kidney failure, thromboembolic events and an increased risk of strokes and heart attacks. Routine calculated antimicrobial treatment is not indicated. In particular, illnesses caused by enterotoxin-producing pathogens such as *Staphylococcus* *aureus*, *Bacillus* *cereus*, *Clostridium* *perfringens* do not require antibiotic treatment. These foodborne illnesses are characterized by vomiting, diarrhea and stomach cramps, which are mostly of short duration and self-limiting. The symptoms are treated by electrolyte and fluid replacement. 

Antibiotic treatment should be given if there is evidence of an invasive infection. Clinical signs are blood and mucus in the feces, fever over 38.5°C, marked decline of the patient’s general condition and high inflammatory parameters in the lab results. The presence of risk factors for severe progression, for example in immunosuppressed patients or patients impaired by multimorbidity, dialysis, old age and neoplastic diseases, may also be an indication for the need for antibiotic treatment. If there is no clinical improvement or in case of special factors such as vascular or joint prostheses (particularly relevant in Salmonella infections) may also be an indication for antibiotic treatment [6].

Antibiotics of choice for the calculated empirical treatment are azithromycin, ciprofloxacin or also (i.v. only) cefotaxime/ceftriaxone (see Table 3 (Tab. 3)) [7].

After detection of the pathogen and determining resistance, the prescribed antibiotics may have to be changed when moving towards targeted treatment (see Table 4 (Tab. 4)).

The use of antibiotics was considered contraindicated in suspected or confirmed cases of enterohemorrhagic *Escherichia* coli (EHEC) infections, as older studies suggested that antibiotics may increase the risk of hemolytic uremic syndrome (HUS) or an increase in the release of toxins (suspected Jarisch-Herxheimer reaction) and may prolong the persistence of the pathogens (for example [8]). Other studies have found that antibiotics are beneficial [9], [10]. Beneficial effects have been reported, amongst others, for chloramphenicol, meropenem, azithromycin, rifaximin and tigecycline [11]. Azithromycin, rifaximin, meropenem and tigecycline were also found to be beneficial in the German EHEC/STEC outbreak in 2011 – they neither increased phage activity or toxin release and improved intestinal pathogen elimination [12]. Toxin production was only significantly increased under ciprofloxacin. Due to the risk of ESBL production in EHEC infections with extra-intestinal or generalized forms, carbapenems such as meropenem are now considered as first-line treatments [13]. 

Diverticulitis as symptomatic diverticulosis is a special case of bacterial gastrointestinal infection, generally requiring surgery. In some forms, such as uncomplicated diverticulitis, which are at risk of a complicated progression (arterial hypertension, chronic kidney disease, immunosuppression), antibiotic treatment is recommended. The basis of the treatment are the same drugs recommended for complicated intra-abdominal infections. Cefuroxime, ciprofloxacin, moxifloxacin, ampicillin/sulbactam, piperacillin/tazobactam are most commonly used [14]. However, any treatment for these polymicrobial infections must be individual, adapted to the patient and planned taking account of the potential risk for multidrug-resistant pathogens. Further information on this issue can be found in chapter 7 [15], for example the section on peritonitis.

Another special case are infections by entero- and cytotoxin-producing strains of *Clostridium* *difficile*. Their prevalence has increased dramatically since the 2000s through the emergence of new hypervirulent variants (especially ribotype 027/NAP-1), as well as the number of severe progressions, associated with treatment failure and a relapse rate of at least 20–25% in the wake of previous treatment regimens. To estimate the severity of an infection with *Clostridium* *difficile* (CDI), the following parameters should be considered: Fever >38.5°C, leukocytosis >15.000x 10^9^/l, left shift >20%, band neutrophiles, hypoalbuminemia <30 g/l, creatinine increase >50% of baseline, lactate elevation ≥5 mmol/l and age >65 years. Established risk factors for recurrence are age >65 years, continuation or renewed antibiotic treatment and previous recurrence. Renal insufficiency and immunosuppression are factors that may be relevant. 

In addition to the treatment of the pathogen, detection of a toxigenic *Clostridium* *difficile* infection (CDI) should possibly also lead to changes to ongoing antibiotic treatment. Colitogenic substances leading to the reduction or eradication of significant parts of the anaerobic gut flora, especially *Bacteroides* *fragilis* and *Prevotella* *intermedia*, should be replaced by less selective substances such as tigecycline. The selection of *Clostridium* *difficile* is driven by those antibiotics that have anaerobic activity and have attracted attention in recent years through frequent use. These include the cephalosporins of groups 2, 3 and 4, clindamycin, carbapenems, fluoroquinolones, trimethoprim/sulfamethoxazole but also penicillin combinations with anaerobic activity [16]. Both metronidazole and vancomycin also proved to be promoters of the selection of vancomycin-resistant enterococci [17].

Treatment of CDI is based on metronidazole, vancomycin, and fidaxomicin, the selection of which is based on the clinical severity, recurrence rate and risk of recurrence (see Table 5 (Tab. 5)) [5]. Initial treatment of mild to severe infections without risk of relapse is still based on metronidazole and vancomycin. Oral application of metronidazole is the treatment of choice for mild to moderate progressions (diarrhea without risk factors for severe progression). Initial treatment of severe CDI or moderate CDI with severe risk factors is with vancomycin, the treatment of a recurrence or CDI with a high risk of recurrence, usually in patients >65 years of age and in patients who did not respond to metronidazole [18], is carried out with fidaxomicin. There is no clear preference for the treatment of the first recurrence, vancomycin and fidaxomicin are competing options. The latter narrow action spectrum substance has advantages in that it does not significantly impact the anaerobic microbiome of the intestine and reduces toxin production. These are reasons why lower recurrence rates were seen in studies compared to vancomycin but with comparably good treatment success [19], [20]. The main indication for fidaxomicin is therefore in the treatment of *Clostridium* *difficile* infection recurrences, in the first recurrence still as an alternative to vancomycin, in the second recurrence as the drug of choice as an alternative to vancomycin with gradual reduction. In addition, there is now proven resistance to vancomycin in European countries, which show that treatment options are decreasing: Rates of 3.1–12% were found in Spain, 15% in Italy and 4% in Denmark [21]. Metronidazole also has relevant resistance rates in Europe: 6.3% in Spain [22], 2.5% in Switzerland, 4.5% in the Czech Republic and 8.7% in Denmark [21]. At least until 2013, no resistance was found in Germany in the 500 isolates tested in the 2013 PEG study [23]. Alternatives to these three preparations, which however were only clinically tested in case series, are rifaximin, which according to studies has resistance rates from 5.1% in hospitalized patients [23] to 40.4% in Germany (compare 0% in Austria and 2.5% in Switzerland) [21] and tigecycline [24]. Teicoplanin is said to be more effective than vancomycin in older data but has never established itself in the treatment of CDI [25], [26]. With fusidic acid, development of resistance was often observed during treatment [27]. 

The use of probiotics is controversial with *Escherichia* *coli* Nissle or preparations with *Saccharomyces* *boulardii*, *Bifidobacterium* spp. and *Lactobacillus* *rhamnosus*. In the case of *Saccharomyces* *boulardii* as a probiotic, fungemia is reported in critically ill oncology patients [28]. A Cochrane analysis concludes that out of 8,014 study patients, only 352 were older than 18 and therefore because of the heterogeneity of studies regarding study endpoints and the substances used, there is currently no recommendation for the treatment of acute infectious gastroenteritis [29]. Although probiotics are not therapeutically successful in CDI, they support adequate antibiotic treatment and can improve the patient’s condition. However, this has so far only been shown convincingly for antibiotic-associated diarrhea [30]. The administration of probiotics cannot sufficiently fulfill the need for reconstitution of the anaerobic flora in the microbiome of the intestine. The “transplantation” of feces in the sense of a fecal microbiome transfer (FMT) is more successful. An analysis of the numerous available case series and case reports shows that methodologically nasogastric instillation has a success rate of only 77%, whereas rectal colonoscopic application has a better success rate of up to 94% [31]. A permanent resolution was achieved in 90% of patients with multiple relapsing CDI who took capsule-enclosed feces for two days [32]. Fecal microbiome transplantation can achieve long-term fecal flora restoration for up to 24 weeks. This experimental treatment has gained attention since a randomized controlled trial in patients with multiple CDI recurrence was prematurely terminated in the Netherlands because after including only 43 patients, fecal transplantation was significantly superior to conventional treatment with vancomycin in terms of response to treatment and recurrence-free response (“sustained response”) [33]. However, the understanding of immunological relationships and the resulting consequences regarding FMT is still incomplete. Also, the labor-intensive production of the transplants has not been standardized to date; it is essential to ensure appropriate extensive exclusion tests regarding infection risks (such as HIV, hepatitis B/C, intestinal pathogens). To date there is neither internationally-binding agreement nor is there a final decision on the approval of transplants as a drug (with the purpose of healing patients) or – as with any transplant – as a medical product [34]. The discussion about the status of the FMT is ongoing in Europe and North America and therefore potentially confronts practitioners, who must invoke case by case healing attempts, with forensic issues. There is currently no evidence for FMT as standard treatment for recurrent CDI, so a general recommendation for clinical practice is not possible. This is seen differently in a European recommendation [35], with reference to the only randomized controlled trial on FMT available to date [32].

In case of a severe clinical picture and strong suspicion of a *Clostridium* *difficile* etiology, calculated treatment should begin empirically. Only with very light symptoms can a delay before initiating causal treatment be possible after discontinuing antibiotic treatment if the diarrhea is subsiding.

## Note

This is the fourteenth chapter of the guideline “Calculated initial parenteral treatment of bacterial infections in adults – update 2018” in the 2^nd^ updated version. The German guideline by the Paul-Ehrlich-Gesellschaft für Chemotherapie e.V. (PEG) has been translated to address an international audience.

## Competing interests

The authors declare that they have no competing interests.

## Figures and Tables

**Table 1:**
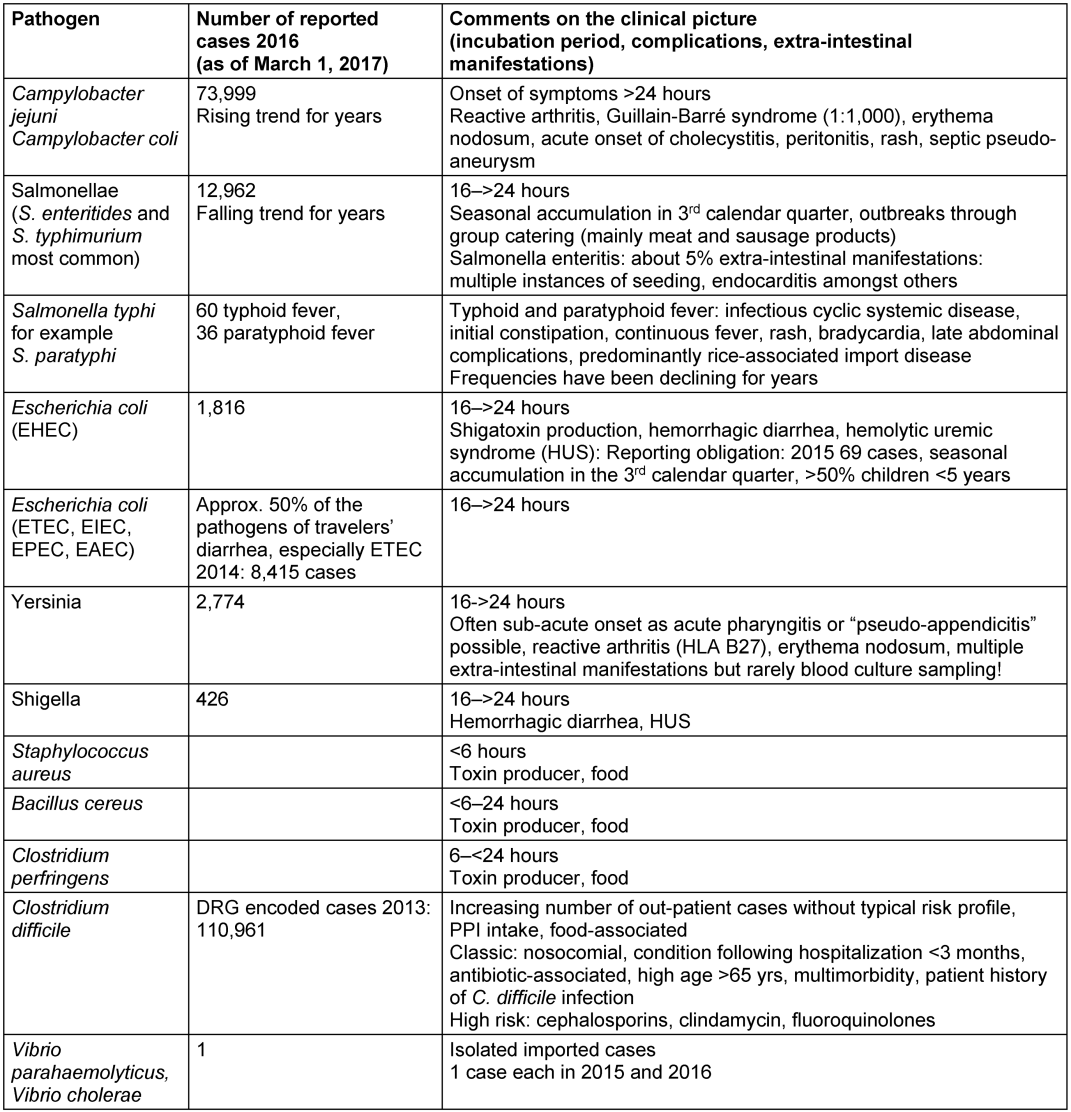
Etiology of bacterial gastrointestinal infections in Germany and their frequencies according to the reporting requirement 2016 [36]

**Table 2:**
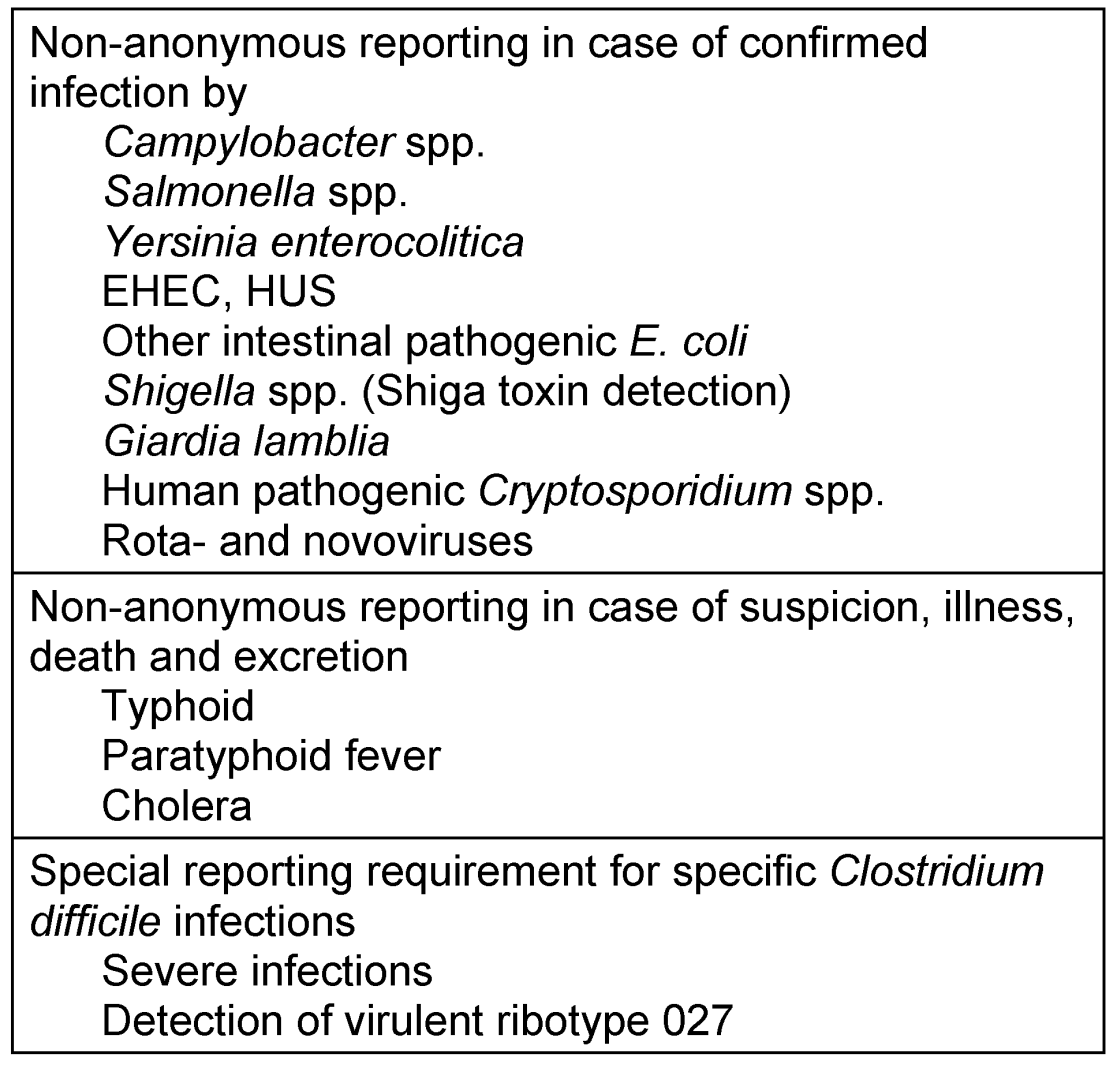
Notifiable gastrointestinal infections

**Table 3:**
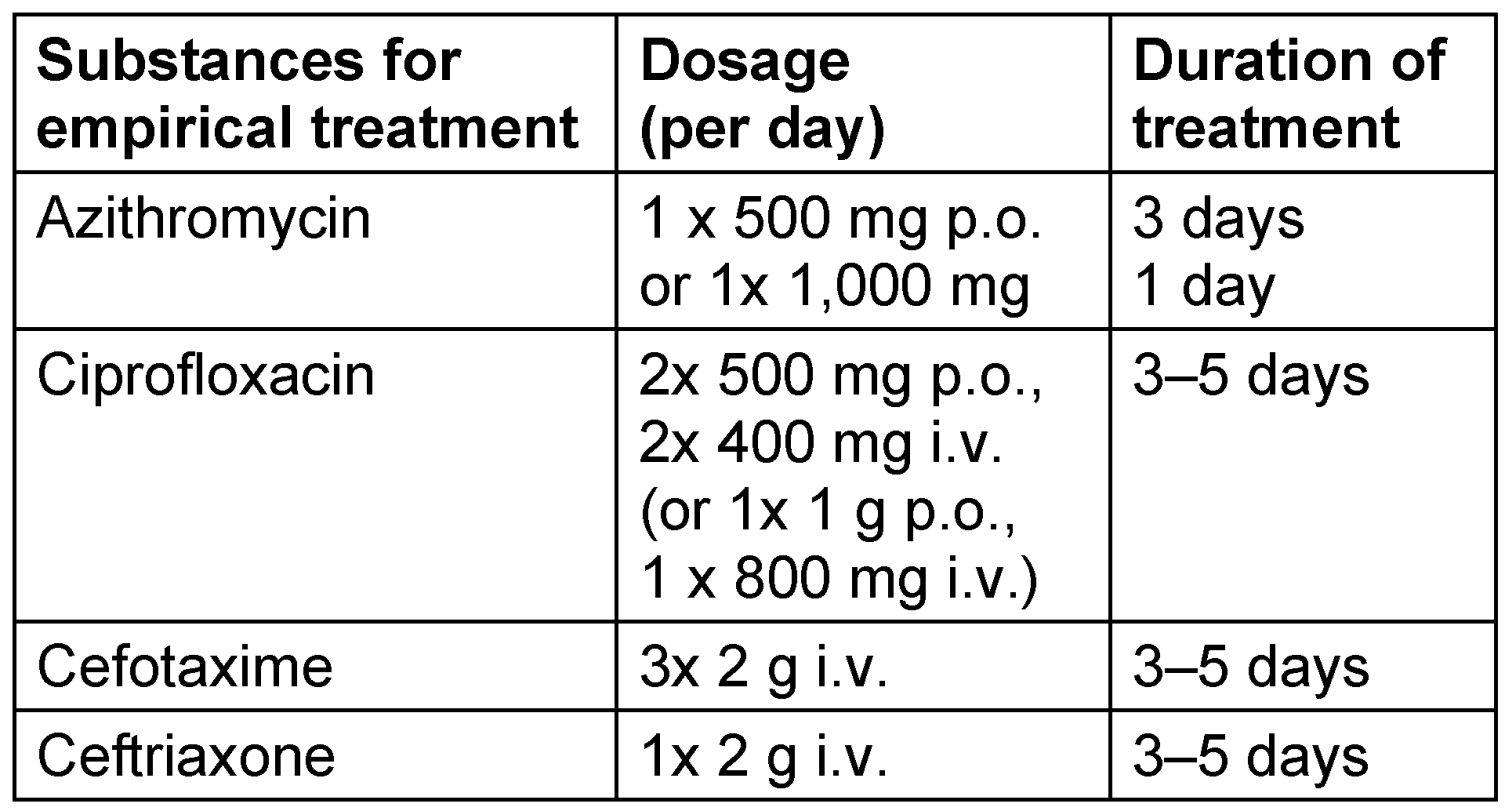
Recommendation for calculated treatment of bacterial gastrointestinal infections

**Table 4:**
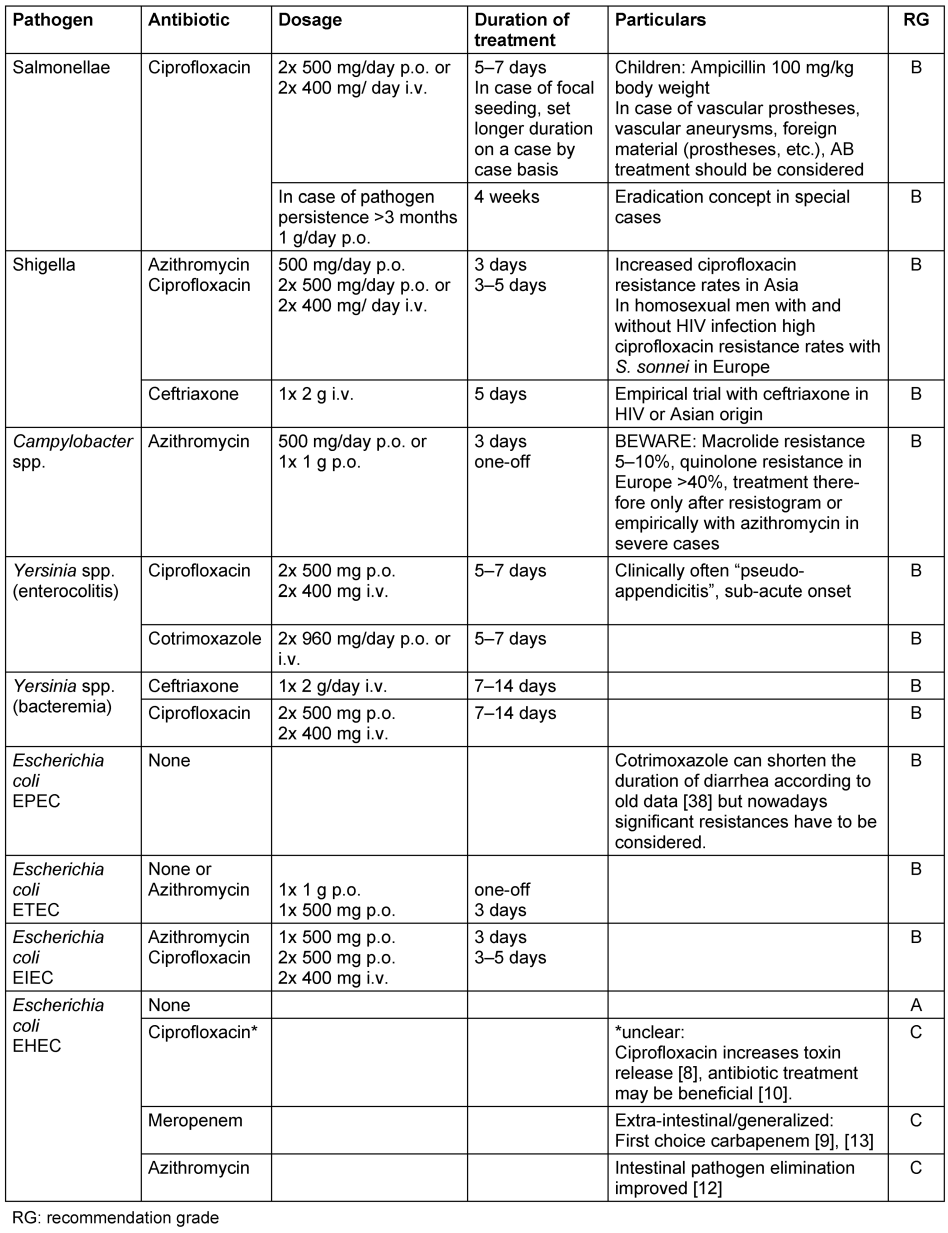
Recommendations for targeted antibiotic treatment in case of known pathogens (according to [7] and [37])

**Table 5:**
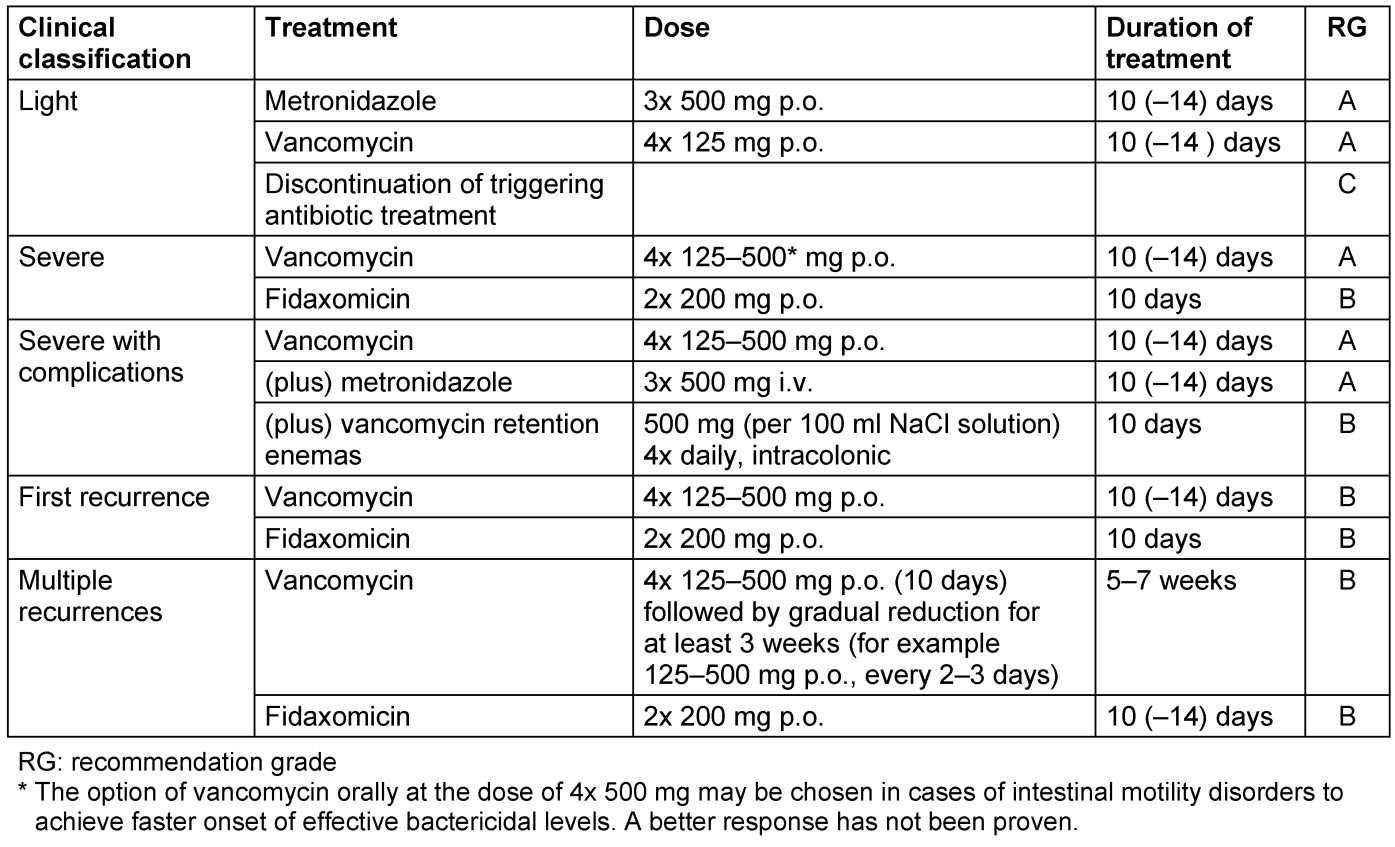
Recommendation for treatment of a *Clostridium difficile* infection [modified after [6], [7], [35])
